# The Parish Doctor

**Published:** 1903-05-16

**Authors:** 


### The Parish Doctor.

We recently received a circular, which we were informed had been pretty widely distributed to the various medical schools and other institutions throughout the United Kingdom, from whose numbers the ranks of the medical profession are recruited. It was intended to make clear for the benefit of future applicants for Poor-law medical appointments in the Highlands and Islands of Scotland what the acceptance of such an office really involves. The circular commences with a table, taken from Local Government Board returns, in which is stated the number of medical officers dismissed by parish councils, since they were instituted in 1895, in the Highland crofting counties. During this period of seven years there were 16 dismissals, and in no fewer than 10 of these cases we are led to understand that no reason was assigned for the removal of the medical men from office, though in the table it is not made perfectly clear whether it was from the Local Government Board, or from the individual most concerned, that the cause of dissatisfaction was withheld. We are reminded that this list does not take note of the large number of medical officers who, owing to petty annoyances and ungenerous treatment, have in their own interests been compelled to resign their appointments, thereby suffering much inconvenience and loss. It is further pointed out that Inspectors of Poor, and all other parish officials, cannot be dismissed without the sanction of the Local Government Board, while in many cases inspectors of poor receive higher salaries, though their duties do not bear comparison with those of the medical officer. During sickness or temporary absence from the parish for a holiday, the parish doctor has to provide a substitute at his own expense, costing him probably four guineas a week, with travelling expenses and board. Other parish officials can get a free and even an extended holiday. In the face of the above facts, all qualified medical men who contemplate applying for parish appointments in the Highlands and Islands of Scotland are advised in their own interests and that of the honour of the medical profession to communicate with the outgoing medical officer in order to be informed what treatment may be expected at the hands of the parish officials, what extra parochial duties may be imposed upon the doctor in respect of his poor-law salary, whether or not he will be called upon to supply drugs and appliances for parish patients at his own expense, and what arrangements will be made for him in case of sickness or in case he requires a holiday.

We are fully in accord with the motives which have led to the distribution of this circular, and welcome it as a sign that the medical profession is becoming awake to the fact that its prestige must suffer unless something is speedily effected towards bettering the position of the parish doctor, not only in the crofting counties of Scotland, but in most rural districts throughout the United Kingdom. It has always been a matter for wonder that medical men have been readily found in sufficient numbers to fill these appointments. When the lot of such a parish doctor is contemplated the only conclusion to which one can arrive is that if the man were not so hard worked he would never be so long-suffering. A miserable pittance is doled out to him—for his salary can by no stretch of words be called a reward—and he in return has to be at the beck and call of every pauper patient in the district by night as well as by day, often has considerable distances to travel, probably has to supply the necessary drugs at his own expense and after all this disposal of time, labour, and mental requirements he is frequently subject to the criticism of those who are considerably his inferiors in the social scale.

A practitioner in a rural district in the South of England kept a record for one year of the distance which he travelled in visiting parish patients and of the working expenses which his parish duties entailed; at the end of the year his nett profit worked out at approximately one halfpenny per mile travelled, and this, without taking account of the value of medical advice given and personal wear and tear brought about by night-work and long hours in the day. If such work as this were undertaken from purely charitable and philanthropic motives it could not fail to arouse the admiration of everyone; but unfortunately it is as a rule taken in hand in order to get and to keep a foothold in the district as a practitioner of medicine, and it is this fact which makes it seem to some minds prejudicial, to say the least of it, to the dignity of the profession at large. It is a pretty well-established law of human nature that the public will hold a man cheaply in their estimation if he places a low value on his labours himself.

